# One Health Approach to Nutritional Status and Well-Being in Food Supply Chain Workers: A Study Protocol

**DOI:** 10.3390/ijerph23010099

**Published:** 2026-01-11

**Authors:** Mariacristina Siotto, Carola Cocco, Chiara Bertoncini, Alessandro Guerrini, Valeria Habib, Erika Antonacci, Elisabetta Ruco, Irene Giovanna Aprile

**Affiliations:** 1 IRCCS Fondazione Don Carlo Gnocchi, 50143 Florence, Italy; ccocco@dongnocchi.it (C.C.); cbertoncini@dongnocchi.it (C.B.); aguerrini@dongnocchi.it (A.G.); vhabib@dongnocchi.it (V.H.); eantonacci@dongnocchi.it (E.A.); eruco@dongnocchi.it (E.R.); iaprile@dongnocchi.it (I.G.A.); 2 Department of Science and Technology for Sustainable Development and One Health, Università Campus Bio-Medico di Roma, 00128 Rome, Italy

**Keywords:** one health, food industry and occupational health, nutritional status, quality of life, mental health, oxidative stress, bioelectrical impedance

## Abstract

**Highlights:**

**Public health relevance—How does this work relate to a public health issue?**

- This protocol is motivated by the high burden of workplace accidents and musculoskeletal disorders among food-supply chain workers, a sector of major importance for public health and economic stability in Italy.
- It targets conditions that are among the leading global causes of disability, long-term functional impairment, and healthcare utilization.

**Public health significance—Why is this work of significance to public health?**

- It applies a multidimensional “One Health” approach that integrates nutritional, psychological, biochemical, and body-composition indicators domains that are rarely assessed together in real-world occupational settings.
- The generation of harmonized and reproducible data will support system-level prevention strategies aimed at reducing avoidable disability, sick leave, and downstream healthcare costs in the food supply chain sector.

**Public health implications—What are the key implications or messages for practitioners, policy makers and/or researchers in public health?**

- The protocol provides an operational framework for the early identification of high-risk worker subgroups, informing targeted nutritional and psychosocial preventive interventions.
- Its multidisciplinary design can assist policymakers and occupational health services in developing integrated surveillance and prevention systems tailored to the food-supply chain, strengthening workforce resilience and public health preparedness.

**Abstract:**

The agri-food supply chain is a relevant contributor to the Italian economy but shows a high incidence of occupational injuries and musculoskeletal disorders, such as lower back pain. Repetitive manual handling and biomechanical overload highlight the need for a prevention-oriented, system-level assessment. This protocol aims to implement a harmonized One Health approach procedure for the multidimensional evaluation of food supply chain workers in real-world settings. The protocol integrates bioelectrical impedance vector analysis (BIVA), nutritional parameters, quality-of-life and psychological measures, and assessments of systemic oxidative stress and systemic serotonin levels. Data from active workers will be compared with those from sedentary individuals. The study will evaluate whether BIVA profiles differ between these groups and examine how the additional indicators contribute to a multidimensional well-being framework. By operationalizing an integrated One Health approach that bridges nutritional, psychological, and biomarker domains, this protocol is designed to guide targeted preventive and educational strategies and inform evidence-based occupational and public health policies across the food supply chain. Trial registration: NCT06896877 (ClinicalTrials.gov), 26 March 2025.

## 1. Introduction

### 1.1. Background

The agri-food supply chain employs a large workforce across primary production, processing, distribution and food services, and represents a structurally relevant domain in terms of value added and employment in Italy [1]. Over the last ten years, the food industry has grown by more than 10%, with a steady and significant increase in the share of exports based on the importance of the “Made in Italy” label.

Despite the operational resilience also documented during emergency scenarios such as COVID-19, this sector remains among those with the highest incidence of occupational injuries and work-related diseases, particularly musculoskeletal disorders [2]. Repetitive manual handling, sustained awkward postures and biomechanical overload persist despite technological advances, and contribute to the upstream burden of conditions such as low back pain, which is the leading cause of disability worldwide [3,4,5]. Consequently, preventive action against these exposures may help reduce the likelihood and severity of disorders, and in turn may contribute to limiting downstream consequences such as prolonged sick leave, hospitalization or the need for rehabilitation.

Given the strategic relevance of the food supply chain in the Italian context, an integrated approach to ensuring the comprehensive protection of workers in this sector is essential. For this reason, the One Health Framework was deliberately adopted as the conceptual foundation of the present study. Defined as a multidimensional model shaped by interdependent human, environmental, and systemic determinants, the One Health Framework aligns with the WHO definition of health as “a state of complete physical, mental and social well-being and not merely the absence of disease or infirmity” [6,7].

Understood as an intrinsically multidimensional model that links human, environmental, and systemic determinants of health, the One Health Framework guides the design, data collection strategy, and analytical plan of this protocol. Its adoption enables a prevention-oriented perspective, as upstream determinants affecting workers’ physical, nutritional, psychological, and biochemical status have implications not only for individual well-being but also for workforce functionality, production stability, and downstream public health.

Despite existing research on ergonomics and mental health in occupational settings, there is a lack of integrated protocols that simultaneously combine nutritional indicators, psychological assessments, and biomarkers within a unified prevention-oriented approach. This study addresses this gap by operationalizing a comprehensive One Health approach that bridges these domains, providing a holistic understanding of worker health that can inform both occupational and public health policy.

Current evidence on agri-food workers remains largely domain-specific: studies typically focus separately on mental health, heat-related risks, or ergonomic exposures, and reviews document fragmented findings by domain [8,9], while no integrated assessments combine nutritional, compositional, psychological and biological dimensions within a unified prevention-oriented framework. Determinants such as nutritional status, body composition, hydration, and emotional well-being influence musculoskeletal resilience and overall health [10,11,12,13].

Beyond nutritional status, quality of life and emotional–affective functioning are fundamental components of worker well-being. Approximately 15% of working-age adults experience a mental disorder during their lifetime [14] and the COVID-19 pandemic produced an estimated 25% increase in anxiety and depression in 2020 [15,16]. Mental health disorders contribute to sick leave and productivity loss, with repercussions for organizational sustainability [17,18]. In recognition of this burden, recent WHO guidelines emphasize the systematic assessment of psychosocial risks and mental health in the workplace through validated instruments on mood, stress, satisfaction and organizational climate [14,19].

In parallel to psychological and behavioural indicators, selected biological markers have been investigated as correlates of altered well-being. Serotonin (5-HT), a tryptophan-derived monoamine regulating circadian rhythms and satiety, has been implicated in mood, anxiety, sleep and cognition. Reduced serotonergic tone has been linked to depressive-related cognitive impairments, including reduced flexibility, memory and attentional control [20,21], and low extracellular 5-HT may compromise memory consolidation [22]. The systemic oxidative state—reflecting the balance between pro-oxidant and antioxidant species—has been associated with inflammatory, respiratory, metabolic, cardiovascular, oncologic, neurodegenerative and anxiety conditions [23,24,25,26].

Body composition is a relevant intermediate indicator linking nutritional and functional status to musculoskeletal risk. Bioimpedance-based assessment provides a non-invasive measure capable of capturing hydration and cellular integrity, which are associated with functional capacity and vulnerability to biomechanical load. Despite its applicability in occupational settings, such measures have not been integrated with psychological, nutritional and biochemical indicators in agri-food workers.

This observational exploratory study aims to generate an integrated assessment of food supply chain workers by combining indicators of nutritional status, bioimpedance-based body composition, psychological well-being, quality of life, and biomarkers of oxidative stress and serotonin. This multidimensional data collection is expected to identify critical issues and modifiable targets, supporting the design of sector-specific preventive and educational strategies.

### 1.2. Aim and Hypothesis

This exploratory observational study primarily aims to assess whether differences in nutritional status and body composition—as indexed by bioimpedance-based assessment of body cell mass and hydration—exist between sedentary and physically active workers in the food supply chain.

As secondary exploratory aims, we will: (i) assess quality of life and emotional–affective well-being using validated instruments, in order to identify psychological or social critical issues related to work in the food supply chain; (ii) characterize nutritional status and dietary habits to detect potential deficiencies or excesses; (iii) quantify systemic biomarkers of oxidative stress and circulating serotonin levels; (iv) identify risk classes, i.e., subgroups of workers at potentially higher disease risk due to inadequate dietary patterns and/or exposure to occupational stressors; (v) assess sex-related differences in nutritional status, oxidative stress biomarkers, and psychological outcomes, to inform the potential need for gender-specific preventive or dietary recommendations; and (vi) analyze the standardized phase angle (SPhA) as an integrative marker of cellular health and systemic status, and explore its potential associations with nutritional, biochemical and psychological indicators on an exploratory basis.

## 2. Experimental Design

### 2.1. Study Design

This study protocol describes a monocentric, observational, non-profit investigation conducted on workers employed in the food supply chain. A total of 40 participants will be recruited from workers directly or indirectly connected with the Experimental Centre “Centro Santa Maria della Provvidenza”, of the Don Carlo Gnocchi Foundation in Rome, Italy. Information about the study will be disseminated to workers through internal communications, corporate informational materials and dedicated dissemination meetings. Participation will be requested by the investigator on a voluntary basis.

Anamnestic data will be collected using Case Report Forms (CRFs). Nutritional status, oxidative stress and serotonin levels will be assessed, and quality of life and emotional–affective status will be evaluated using validated questionnaires. Moreover, surveys covering general, nutritional, psychological and occupational health domains will be administered in subsequent study phases for exploratory purposes. An informational brochure promoting healthy dietary practices will be delivered to all participants during the evaluation phase.

**Figure 1:**
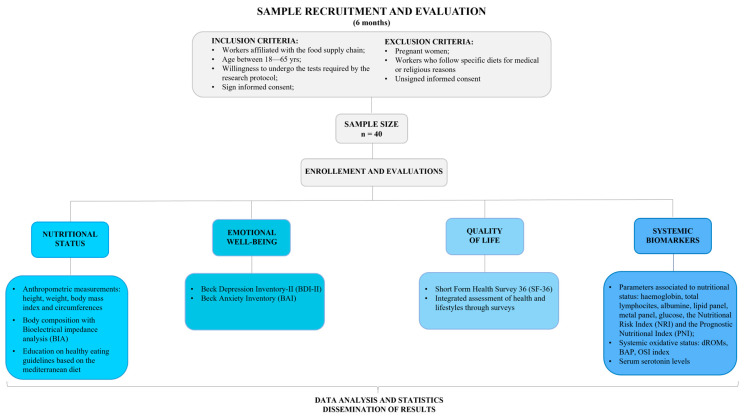
Flow chart of the observational protocol study.

Figure 1 shows the study flow chart, in compliance with the Centralised Protocol Items Recommendations for Observational Studies (SPIROS) guidelines (see Table S1).

The total duration of the study will be six months, with completion expected by 30 November 2025. During this period, specific days will be scheduled to convene participants, during which all assessments planned in the protocol will be conducted in a single session (T0). At this time, participants will be provided with the link to access and complete the online surveys. The study protocol is registered at ClinicalTrials.gov ID: NCT06896877. The study protocol is registered at ClinicalTrials.gov under the name “Nutrition status and emotional well-being of the worker-consumer in the food supply chain. (ONEHEALTH)” on 26 March 2025 and ID: NCT06896877.

### 2.2. Participant Selection for Cohort Study

The patient sample will be recruited at the recruitment centre from workers directly affiliated with the Trial Centre or indirectly connected to it as part of the food supply chain. Patient screening will be carried out by a multidisciplinary team and will aim to determine the subject’s eligibility according to the following inclusion and exclusion criteria.

Inclusion criteria: (i) workers affiliated with the Trial Centre or indirectly connected to the Trial Centre as part of the food supply chain; (ii) adults aged between 18 and 65 years; (iii) willingness to undergo specific tests: assessments of nutritional status, investigations into quality of life and emotional–affective wellbeing; (iv) signed informed consent.

Exclusion criteria: (i) pregnant women will be excluded to avoid physiological changes related to pregnancy affecting the measurements; (ii) workers following specific diets for medical or religious reasons, which may not be representative of the general food supply chain population, will be excluded; (iii) unsigned informed consent.

In addition to excluding pregnant women and individuals following medically prescribed diets, information on chronic medical conditions and medications known to influence serotonin metabolism, oxidative stress, inflammatory status, or body composition will be systematically collected. Rather than excluding these participants, such variables will be treated as potential confounders and incorporated into the analytical strategy (e.g., sensitivity analyses, adjusted models) to maintain ecological validity while minimizing biomarker bias.

Once eligibility has been assessed, participants will be provided with an informed consent form, which they will be required to read and sign before being formally enrolled in the study. The entire process will be overseen by the principal investigator, who will ensure that participants are properly informed and that their voluntary participation is respected, in accordance with the procedures described below.

### 2.3. Data Management

As both Promoter and Trial Centre, the Don Gnocchi Foundation retains exclusive ownership of the data collected during the study, in accordance with Regulation (EU) No. 536/2014 [27] and Ministerial Decree of 30 November 2021 [28].

Upon obtaining informed consent, data will be initially collected on paper Case Report Forms (CRFs) and subsequently entered in pseudonymized form into a secure electronic database by the investigator.

Specifically, each participant will be assigned a unique alphanumeric identification code (Subject ID). The correspondence between the participant’s personal data and the Subject ID will be recorded in a dedicated “Subject Identification Log”, accessible only to the principal investigator and authorized collaborators. This document will be destroyed upon completion of the study. Data collection will be carried out by the Trial Centre, and data will be entered into the electronic database in a pseudonymized format. The principal investigator will oversee quality assurance procedures. Pseudonymization will be performed in such a way that individuals accessing the database cannot, under any circumstances, trace the identity of the study participants. Only the principal investigator and authorized collaborators will have access to the information required to re-identify participants enrolled at the Trial Centre.

### 2.4. Data Confidentiality Statement

The data of participants involved in the research project will be processed in full compliance with the right to privacy, in accordance with Regulation (EU) No. 2016/679 (General Data Protection Regulation–GDPR) [29].

Information relating to clinical, demographic, and genealogical data will be collected, processed, stored, and used for the purposes of diagnosis, preventive investigation, and care, ensuring appropriate confidentiality and compliance with privacy legislation (Regulation (EU) No. 2016/679–GDPR [29] and the General Authorisation for the Processing of Genetic Data No. 8/2016 issued by the Italian Data Protection Authority on 15 December 2016) [30].

### 2.5. Ethical Consideration

The study will be conducted in accordance with Italian legislation and in compliance with the principles of the Declaration of Helsinki. Relevant ethical code references include (1) Helsinki: Articles 4, 5, 7; (2) Helsinki: Article 6; (3) Helsinki: Articles 9, 10, 11.

All activities will be carried out in accordance with the provisions of European Regulation 2017/745 [31] and the subsequent Italian Legislative Decree of 5 August 2022, No. 137, as updated on 12 April 2023 [32].

The principal investigator confirms adherence to the procedures described in this protocol and to the principles of Good Clinical Practice (GCP). The study sponsor ensures the protection of all clinical and non-clinical personal data collected during the project, in compliance with national data-protection regulations (D. Lgs. 196/2003) [33].

It is the responsibility of the investigators, or personnel delegated by them, to obtain informed consent after providing participants with clear and complete information on the study aims, procedures, anticipated benefits, and potential risks. Participants must also be explicitly reassured that refusal to participate or withdrawal at any time will not result in any disadvantage. The informed consent form includes all details necessary for participants to give free and reasoned consent. Before signing, each participant will be invited to read the form carefully. The accompanying information sheet outlines the assessments to be performed, any risks associated with them, and the pseudonymization procedures applied to protect personal identity. Pseudonymization allows re-identification only in two situations: (i) when findings relevant to a participant’s health emerge and the individual has authorized recontact for this purpose; and (ii) when the participant requests the deletion of their personal data from the study database.

The study protocol underwent ethical review by the local ethics committee, Comitato Etico Territoriale Lazio Area 1, and received approval on 20 February 2025.

## 3. Procedure

**Figure 2:**
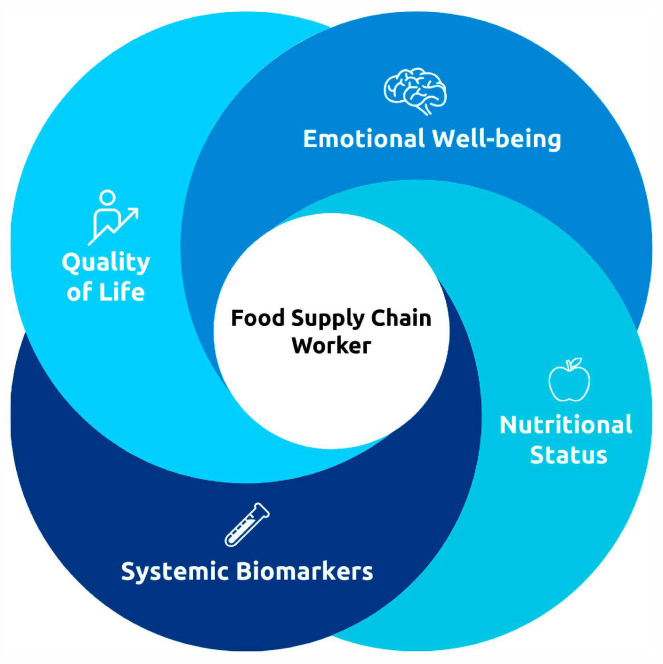
Overview of the multidisciplinary and multidimensional assessment framework of food supply chain workers, integrating validated measures of quality of life and emotional–affective functioning, nutritional status—comprehensive of an accurate body composition analysis—and selected biological markers.

The evaluation of worker well-being will integrate validated measures of quality of life and emotional–affective functioning with nutritional and physical indicators, together with selected biological markers, as shown in Figure 2.

### 3.1. Anamnestic and Lifestyle Information

Once enrolled, the following data will be recorded for each participant: demographic and general health information and smoking habits; use of medications, supplements and nutraceuticals; and type of work and number of working hours per day, week and year and active breaks during work.

### 3.2. Nutritional Status

#### 3.2.1. Anthropometric Measurement Assessment

Anthropometric measurements will include assessments of height and body weight. Weight will be assessed to an accuracy of 0.1 kg on a calibrated scale. Measurements will be taken in the morning after an overnight fast, without heavy clothing or shoes. Height will be recorded and reported in metres (m). Body mass index (BMI) at T0 and T1 will be calculated, expressed in kg/m^2^, and circumferences (arm, calf, waist and hips) will be measured and reported in centimetres (cm). The waist-to-hip ratio (WHR) will also be calculated.

#### 3.2.2. Body Composition Assessment

Body composition assessment will be assessed using a tetrapolar bioelectrical impedance analysis (BIA) at a single frequency of 50 kHz (101 anniversary sports edition, Akern s.r.l., Florence, Italy). Bioelectrical impedance analysis (BIA) was performed using four certified electrodes (BIATRODES, Akern, Florence, Italy), arranged in two pairs positioned 5 cm apart on the metacarpal and metatarsal regions of the non-hemiparetic side. Measurements were taken with participants lying supine and with limbs positioned symmetrically. Prior to electrode placement, the skin was cleaned to ensure optimal conductivity and reduce potential artefacts. Individuals with fever or hypothermia were not assessed, as body temperature can influence electrical resistance. Hydration status was clinically evaluated before conducting BIA, and participants showing marked dehydration or peripheral edema were excluded. Subjects with implanted pacemakers were also not eligible. All operators received specific instructions to ensure correct data acquisition and minimize potential measurement errors.

Raw bioelectrical data consisted of Resistance (R, Ω) and Reactance (Xc, Ω) acquired under standardized conditions. These raw measures were exported and processed using the Bodygram^®^ analysis software (v. 3.3.19, Akern, Florence, Italy), which automatically computes derived impedance parameters according to the manufacturer’s validated algorithms (tissue hydration percentage, nutritional index, resistance, reactance, phase angle, standardized phase angle (SPhA), total body water, extracellular water, intracellular water, lean mass, fat mass, body cell mass, musculoskeletal mass, appendicular muscle mass, basal metabolic rate, and total daily energy expenditure).

Bioelectrical Impedance Vector Analysis (BIVA) will also be derived from raw data. Specifically, R and Xc were standardized by height (H, m) to obtain R/H and Xc/H, and the resulting values were plotted on the R–Xc plane to generate individual impedance vectors. The software provides BIVA graphical output as well as derived indices (e.g., vector length and phase angle), enabling subsequent group-level comparison of vector position. For an extensive description of BIVA, see Guerrini et al. [34].

#### 3.2.3. Eating Habits Questionnaire

The participant will also complete a survey on dietary habits, including daily water intake and consumption of other beverages (including alcoholic drinks), adherence to the Mediterranean diet (weekly intake of olive oil, nuts, cereals and whole grains, vegetables, meat, dairy products, processed and industrial foods, chocolate, coffee and tea), as well as the presence of specific dietary regimens and eating habits.

#### 3.2.4. Healthy Eating Education

To promote healthy dietary practices, during the evaluation, we will deliver an informational brochure providing guidance for a healthy lifestyle, based on the principles of the Mediterranean diet and in accordance with the criteria outlined by the National Recommended Energy and Nutrient Intake Levels for the Italian Population [35]. In the brochure, there is a link to the dietary Guidelines from CREA-AN [36]. These guidelines are part of a multifaceted solution to encourage health and prevent diet-related chronic diseases, including cardiovascular disease, type 2 diabetes, some cancers, and obesity. It is a consensus document accepted and endorsed by the scientific community and civil society stakeholders [37].

### 3.3. Systemic Biomarkers Analysis

All the biological sample processing and analyses will be conducted at the Biochemistry and Molecular Biology Research Laboratory located within the trial centre. Upon completion of the analyses foreseen in the project, the samples will be destroyed.

Approximately 20 mL of venous blood will be collected in the morning following an overnight fast. Whole blood will be processed immediately for standard hemochromocytometric parameters. Serum will be separated by centrifuging the samples at 3000 rpm for 15 min and subsequently stored at −80 °C under controlled conditions until laboratory analyses are performed. Serum will be thawed immediately before analyses and will be analyzed in duplicate.

#### 3.3.1. Hematochemical Parameters Associated with Nutritional Status

Whole blood analysis will include hemochromocytometric indices (hemoglobin and total lymphocyte count) using a photo-impedance fluorescence cytometmethod. Moreover, the following analysis will be performed by testing every parameter on an integrated analytical photometer (Diacron International S.r.l., Grosseto, GR, Italy):

- Glucose was assessed by an oxidase/peroxidase system [38].
- Total cholesterol measured by means of oxidation from a cholesteroxidase to cholest-4-en-3-one [39].
- Direct HDL cholesterol assessed by a transformation of the HDL portion into a quinone derivative [40].
- Triglycerides measured with a peroxidase-coupled method [41].
- Calcium was analyzed using a method based on the o-cresolphthalein complexone test [42].
- Magnesium was assessed using a direct colorimetric assay based on the xylidyl Blue-I method [43].
- The Prognostic Nutritional Index (PNI) is a tool used to assess the immunological and nutritional status of patients, particularly in predicting surgical outcomes, cancer prognosis, and postoperative complications. The PNI is calculated using the following formula: PNI = (10 × serum albumin (g/dL)) + (0.005 × total lymphocyte count per mm^3^). The lower the score, the higher the risk (normal nutritional status, low risk: PNI > 38; moderate risk: scores between 35 and 38 severe malnutrition, high risk: PNI < 35).
- The Nutritional Risk Index (NRI) is a tool designed to assess a patient’s nutritional status and predict the risk of complications associated with malnutrition. It is calculated using the following formula: NRI = (1.519 × serum albumin [g/L]) + (41.7 × current weight [kg]/usual weight [kg]). The lower the score, the higher the risk (no risk: NRI > 100; severe risk: NRI < 83.5).

#### 3.3.2. Assessment of Biomarker of Systemic Oxidative Status

The systemic oxidative status will be analyzed through the following analyses on an integrated analytical photometer (Diacron International S.r.l., Grosseto, GR, Italy).

- Total hydroperoxides colorimetric determination of ROMs (Reactive Oxygen Metabolites) (d-ROMs test; Diacron International S.r.l., Grosseto, GR, Italy). In the d-ROMs test, ROMs (primarily hydroperoxides) contained in the biological sample, close to iron, generate alkoxyl and peroxyl radicals, according to the Fenton’s reaction. The radicals, reacting with a chromogenic mixture, oxidize it and transform it into a photometrically measurable coloured derivative. The measurements are reported in arbitrary units (UCARR), where 1 UCARR corresponds to 0.08 mg/100 mL of hydrogen peroxide [40]. Values between 250 and 300 UCARR fall within the normal reference range; 301–320 UCARR are classified as borderline; 321–340 UCARR indicate low oxidative stress; 341–400 UCARR indicate moderate oxidative stress; 401–500 UCARR reflect high oxidative stress; and values exceeding 500 UCARR correspond to very high oxidative stress (Diacron International S.r.l., Grosseto, GR, Italy). The reproducibility of the test reported as CV% is 1.72.
- The total biological antioxidant capacity (BAP-Test; Diacron International S.r.l., Grosseto, GR, Italy) quantifies the overall pool of endogenous (e.g., bilirubin, uric acid, proteins) and exogenous (e.g., ascorbate, tocopherols, carotenoids, flavonoids) plasma components capable of exerting antioxidant activity and neutralizing reactive radical species. In the BAP test, the biological antioxidant potential of plasma barrier is based on the ability of all antioxidants active to reduce a coloured solution of ferric ions (Fe^3+^) to ferrous ions (Fe^2+^), resulting in a decolouration of the solution that is photometrically detectable. Reference values in µmol/L that are >2200 are intended as “normal antioxidant status”, 2200–2000 are considered a “border line antioxidant status, 2000–1800 are interpreted as “at slight antioxidant deficiency status”, 1800–1600 as “deficiency of antioxidant status”, <1600 as a “high and very high deficiency of antioxidant status” condition (Diacron International S.r.l., Grosseto, GR, Italy). The reproducibility of this test expressed as CV% is 2.32.
- Calculation of the OSI index to assess the relative antioxidant capacity, namely, the antioxidant capacity normalized for total circulating hydroperoxides. This index is the antioxidative/oxidative stress ratio assessed by the ratio equation: BAP/d-ROMs. We refer to this ratio as the OSI index, which reflects the system’s potential antioxidant capacity [44]. A threshold value of 7.3 was adopted for the BAP/d-ROMs ratio; values below this cut-off were classified as “oxidised,” whereas values equal to or above 7.3 were considered “reduced” [45].

#### 3.3.3. Assessment of Serotonin

Serum serotonin concentrations will be quantified using a fast-track ELISA immunoassay (Mybiosource, San Diego, CA, USA) and read on an absorbance microplate reader (iMARK™, BIO-RAD Laboratory Inc., Hercules, CA, USA). The assay involves an initial quantitative acylation step, followed by a competitive reaction that enables detection at 450 nm. Reference values for serum serotonin range from 70 to 270 ng/mL.

### 3.4. Assessment of Quality of Life

The SF-36 is a self-administered questionnaire designed to assess both physical and mental health-related quality of life (HRQoL). It consists of 36 items grouped into eight scales: Physical Functioning (PF), Physical Role (PR), Bodily Pain (BP), General Health (GH), Vitality (VT), Social Functioning (SF), Emotional Role (ER), and Mental Health (MH). Each scale is rated from 0 to 100, with higher scores indicating a better subjective perception of one’s health. The SF-36 also produces two summary scores: the Physical Component Summary (PCS) and the Mental Component Summary (MCS), which represent overall physical and psychological well-being [46,47].

### 3.5. Assessment of Emotional–Affective Sphere

In this study, widely used and validated instruments were selected from the literature, which have proven to be sensitive and reliable in detecting the constructs under investigation [48,49]. The choice was guided by the need to balance psychometric accuracy and speed of administration, as the instruments are included among numerous other assessment procedures.

In particular, the Beck Depression Inventory-II (BDI-II) and the Beck Anxiety Inventory (BAI) were chosen for their ability to measure depressive and anxiety symptoms in a specific and distinct manner. The BAI, in particular, was constructed to include mainly physiological and somatic symptoms, minimizing overlap with the affective dimensions typical of depression. In addition, the Short Form Health Survey-36 items (SF-36) provides a complementary perspective, allowing the overall impact of symptoms (physical or psychological) on daily functioning to be assessed. This tool allows the analysis to be extended to the domain of perceived quality of life, including physical, psychological, and social components, in line with an integrated approach to worker health. Below is a specific description of each tool mentioned:

(i) The BAI is a paper-and-pencil self-report or interviewer-administered questionnaire that can be administered in an individual format. It allows the severity of anxiety symptoms in adults to be assessed. The scale consists of 21 items, each of which describes a common symptom of anxiety. Respondents are asked to rate how much each symptom has bothered them in the past week on a 4-point Likert scale ranging from 0 (“not at all”) to 3 (“severely”). The total score provides an index of anxiety severity, with higher scores indicating greater symptom intensity [48,50].

(ii) The BDI-II is a self-administered tool consisting of 21 items designed to assess the severity of depression in adults and adolescents aged 13 and older. It is based on the criteria for the diagnosis of depressive disorders listed in the fourth edition of the Diagnostic and Statistical Manual of Mental Disorders (DSM-IV) of the American Psychiatric Association [51]. The scale consists of 21 items scored on a 4-point Likert format (0 = no symptoms, 3 = severe symptoms). It assesses two main domains: somatic–affective manifestations (12 items, e.g., reduced pleasure, low energy, fatigue) and cognitive features (9 items, e.g., self-esteem, self-criticism, feelings of worthlessness). Finally, the BDI-II yields a total score reflecting the overall severity of depressive symptoms [49,50].

### 3.6. Sample Size and Statistical Analysis

This is an exploratory, two-group study (active workers vs. sedentary workers). We planned a total sample of 40 participants (≈20 per group). A priori calculations were performed with G*Power (v3.1.9.7) to inform feasibility for a two-group multivariate comparison approximating Hotelling’s T^2^ (implemented as a MANOVA with two dependent variables, R/H and Xc/H). Under conventional assumptions (two-sided α = 0.05; target power ≈ 0.80; small-to-moderate multivariate effect), a sample size of 35 is typically adequate; therefore, we set N = 40 to accommodate measurement variability and potential attrition while maintaining balanced groups. Although the sample size calculation is fixed to the primary bioimpedance outcomes, the nutritional, biochemical, and psychological variables will be treated as exploratory secondary outcomes. For these, the study aims to estimate effect sizes and variability and to evaluate feasibility rather than to achieve confirmatory statistical power.

Raw R and Xc will be standardized by height (H) to obtain R/H and Xc/H. For each group, we will compute the mean vector and its 95% confidence ellipse on the RXc plane. The primary comparison between active vs. sedentary workers is the displacement of the mean impedance vector assessed with two-sample Hotelling’s T^2^ (reporting T^2^, *p*-value, Mahalanobis distance, and 95% CI for the mean-vector difference). Visualization will include the RXc-mean graph with group ellipses. Covariate-adjusted MAN(C)OVA models will be used as sensitivity analyses to assess robustness. In addition to age and sex—variables inherently related to BIVA interpretation—analyses will adjust for occupational factors (e.g., type of work tasks, years in role, ergonomic load) and lifestyle habits (e.g., smoking, alcohol consumption, physical activity outside work), which may influence hydration, body composition, metabolic state, or psychological indicators. Contemporary reference/tolerance ellipses for interpretation will follow Campa et al. [52] or the most appropriate sex-/age-specific set for our sample.

Standardized phase angle (SPhA), adjusted to sex- and age-specific references, will be analyzed as a continuous endpoint. The primary comparison (active vs. sedentary) will use a two-sample t-test. A sensitivity ANCOVA adjusting for BMI (and other prespecified covariates, if applicable) will assess robustness. The proportion of participants with SPhA ≤−1.65 (≈5th percentile) will be compared between groups using χ^2^ or Fisher’s exact test (reporting risk ratio and 95% CI). Secondary exploratory outcomes, deriving from nutritional, biochemical, and psychological domains’ assessment, will be analyzed with χ^2^/Fisher for categorical variables and t-test/Mann–Whitney for continuous variables depending on normality (assessed with Shapiro–Wilk). Effect sizes with 95% confidence intervals will be reported. Age will be included as a continuous covariate in sensitivity MAN(C)OVA and ANCOVA models. Exploratory analyses will also examine age-related associations with bioimpedance, psychological, and biomarker measures.

Exploratory associations among nutritional indicators (SPhA), bioimpedance parameters, oxidative-stress markers, serotonin, and psychological measures will be examined using Pearson or Spearman coefficients as appropriate. No directional hypotheses are pre-specified for these exploratory analyses; findings will be interpreted as hypothesis-generating.

No missing data are expected because all measurements will be collected during supervised in-person sessions. Data completeness will be verified at the time of assessment. If unexpected missingness occurs, analyses will follow a complete-case approach and the extent of missing data will be reported.

## 4. Expected Results

We expect active workers to show body-impedance vector profiles compatible with greater cellular integrity and fluid balance compared to sedentary workers, interpreted phenomenologically and without causal claims. In nutritional, biochemical, and psychological domains, we anticipate heterogeneous patterns and weak, non-directional associations, reflecting multidimensional variability across activity categories. Descriptive estimates and their uncertainty will be reported for all variables, and any analyses not pre-specified in the protocol will be clearly labelled as exploratory. If multiple exploratory tests are conducted, procedures to limit false-positive findings will be applied.

To strengthen the translational value of the study, we anticipate that nutritional indicators (e.g., low SPhA or hydration-related shifts in BIVA) may help identify workers who could benefit from targeted dietary support or hydration-related interventions. Psychological and quality-of-life measures may highlight groups experiencing elevated stress, low mood, or reduced well-being, supporting initiatives such as stress-management programmes or workplace climate interventions. Biochemical markers, including oxidative stress and systemic serotonin levels, may reveal upstream imbalances that can inform tailored health promotion strategies—ranging from programmes addressing fatigue and sleep quality to initiatives supporting mental health and emotional regulation.

Potential at-risk profiles emerging from these multidomain patterns will be flagged only to inform future hypothesis-driven prevention, not as pre-specified outcomes of this study. A One Health perspective will orient the interpretation of findings toward upstream determinants by jointly considering human, organizational, and environmental factors in the agri-food supply chain. This multidisciplinary protocol aims to demonstrate the feasibility of an integrated assessment framework capable of guiding sector-specific preventive and educational strategies.

## 5. Discussion and Conclusions

Multidimensional differences in body composition, nutritional status, and psychological well-being have been documented in occupational settings and may reflect upstream determinants of health that interact across physical, organizational, and environmental domains. By adopting a One Health Framework, the present protocol integrates these determinants into a unified assessment strategy, providing a conceptual basis for identifying emerging risk profiles and informing preventive actions. The study will generate feasibility data and preliminary indications of how multidomain assessments can support targeted interventions, including ergonomic optimization, mental-health promotion, and nutritional risk reduction.

The expected impact includes improved identification of modifiable determinants, enhanced prioritization of preventive strategies, and the potential to inform occupational and public health policies tailored to the food supply chain. This is particularly important in a sector where work-related diseases—especially musculoskeletal disorders—remain highly prevalent and often progress to long-term functional limitations and rehabilitation needs. Enhancing preventive measures at an early stage may help decrease both incidence and severity, thereby reducing downstream impacts, including prolonged sick leave and hospitalization.

## Data Availability

The data that support the findings of this study, including the full study protocol, statistical analysis plan, and relevant study materials (e.g., questionnaires and informed consent forms), are available from the corresponding author upon reasonable request. Participant-level data are not publicly available due to privacy and ethical restrictions.

## Supplementary Materials

The following supporting information can be downloaded at https://www.mdpi.com/article/10.3390/ijerph23010099/s1, Table S1: SPIROS 2023 Checklist: Recommended Items to address in the observational study Protocol and related documents.
